# Independent control of natural killer cell responsiveness and homeostasis at steady-state by CD11c+ dendritic cells

**DOI:** 10.1038/srep37996

**Published:** 2016-12-01

**Authors:** Thuy Thanh Luu, Sridharan Ganesan, Arnika Kathleen Wagner, Dhifaf Sarhan, Stephan Meinke, Natalio Garbi, Günter Hämmerling, Evren Alici, Klas Kärre, Benedict J. Chambers, Petter Höglund, Nadir Kadri

**Affiliations:** 1 Center for Hematology and Regenerative Medicine (HERM), Department of Medicine Huddinge, Karolinska Institutet, Stockholm, Sweden; 2 Department of Microbiology, Tumor and Cell Biology (MTC), Karolinska Institutet, Stockholm, Sweden; 3 Department of Oncology-Pathology, Karolinska Institutet, Stockholm, Sweden; 4 Institute of Experimental Immunology, University of Bonn, Germany; 5 German Cancer Research Center DKFZ, Division of Molecular Immunology, Heidelberg, Germany; 6 Department of Medicine, Center for Infectious Medicine, F59, Karolinska Institutet, Karolinska University Hospital Huddinge, Stockholm, Sweden

## Abstract

During infection and inflammation, dendritic cells (DC) provide priming signals for natural killer (NK) cells via mechanisms distinct from their antigen processing and presentation functions. The influence of DC on resting NK cells, i.e. at steady-state, is less well studied. We here demonstrate that as early as 1 day after DC depletion, NK cells in naïve mice downregulated the NKG2D receptor and showed decreased constitutive phosphorylation of AKT and mTOR. Subsequently, apoptotic NK cells appeared in the spleen concomitant with reduced NK cell numbers. At 4 days after the onset of DC depletion, increased NK cell proliferation was seen in the spleen resulting in an accumulation of Ly49 receptor-negative NK cells. In parallel, NK cell responsiveness to ITAM-mediated triggering and cytokine stimulation dropped across maturation stages, suggestive of a functional deficiency independent from the homeostatic effect. A role for IL-15 in maintaining NK cell function was supported by a gene signature analysis of NK cell from DC-depleted mice as well as by *in vivo* DC transfer experiments. We propose that DC, by means of IL-15 transpresentation, are required to maintain not only homeostasis, but also function, at steady-state. These processes appear to be regulated independently from each other.

Dendritic cells (DC) are innate sentinels of the immune system that process and present foreign antigens to T cells^1^. In addition to this role, DC have been shown to provide homeostatic support to naïve T cells, securing their sensitivity to subsequent challenges with cognate antigens^2^,^3^,^4^. A role for DC in NK cell activation and priming has also been suggested^5^,^6^,^7^,^8^,^9^. A question that has so far not been extensively studied, however, is if DC provide basic support for NK cells also at steady-state. Some support for such a role has come from experiments using NK cell adoptive transfer setups or bone marrow chimerice mice^9^,^10^,^11^. In addition, *in vivo* imaging studies, both on tissue sections and intravitally, have demonstrated frequent interactions between NK cells and DC in lymph nodes and in the spleen^12^,^13^, suggesting that NK cells may receive supporting signals from DC at steady-state.

The notion that DC may support resting NK cells is important for the understanding of NK cell biology and for the development of novel therapeutic principles. To study this question critically, timely and well-controlled systems of DC depletion are required. CD11c-DTR mice, in which all DC expression the diptheria toxin receptor (DTR), has demonstrated that DC depletion indirectly affect NK cell function during inflammatory responses. However, these mice are not directly useful in longitudinal studies of DC depletion, because they do not tolerate repeated diphteria toxin (DT) injections^14^. This limitation has forced investigators to use bone marrow chimeric mice and models of adoptive transfer of NK cells in studies of these questions. While results from such studies have supported a regulatory role of DC in NK cell homeostasis, irradiation as such, the existence of radioresistant DC in chimeric mice, and the requirements for lymphopenia to allow studies of adoptively transferred NK cells, complicate the interpretation of the results^10^,^11^,^15^,^16^,^17^.

By using CD11c.DOG mice, in which DC can be selectively depleted for longer time periods without toxicity, we have circumvented these limitations. Using these mice, we provide a comprehensive picture of the molecular and cellular events taking place in the NK cell population after acute DC ablation and up to a time period of 10 days. Our data confirm the notion that NK cells require DC at steady-state to maintain homeostasis. We also show, unexpectedly, that NK cell function is rapidly lost after DC depletion. Both these mechanisms appear to be dependent of IL-15, but follow different kinetics and may be regulated via different pathways. Our data support the existence of a common control mechanism between NK cells and T cells, in which DC interactions guarantee the maintenance of a tonic state of responsiveness in a stage preceeding stimulation of effector responses.

## Results

### Dendritic cells control NK cell homeostasis and maturation at steady state

**Figure 1:**
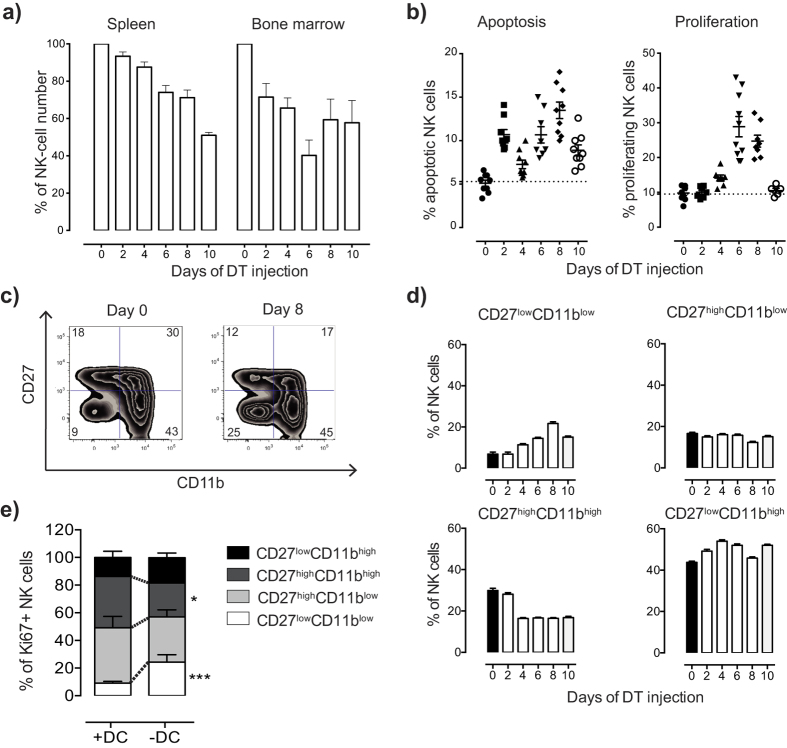
Dendritic cells regulate NK cell homeostasis and maturation status. (**a**) Kinetic analysis of NK cell numbers in spleen and bone marrow. (**b**) Kinetic of NK cell apoptosis (Annexin V^+^ Vivid^+^) (left panel) and proliferation (Ki67^+^) in the spleen. (**c**) Maturation of splenic NK cells at the start (Day 0) and at 8 days after the onset of DT administration (Day 8). (**d**) A kinetic analysis of NK cell maturation in the spleen analyzed by a combination of markers for CD27 and CD11b. (**e**) NK cell maturation status on proliferating (Ki67^+^) cells in the presence or absence of DCs for 6 days.*p < 0.05, ***p < 0.001 (unpaired two-tailed Student’s t-test). Data shown are combined from 2 independent experiments (**a,b,d**), representative of 2 experiments (**c,e**). Error bars, SEM ([n = 5–10] mice per group [**a,b,d**], 5 mice per group [**e**]).

Our first objective in this study was to test if removal of DC over a longer period would affect NK cell homeostasis, and if so, to determine the kinetics of this effect. We first confirmed that DT administration led to an almost complete depletion of CD11c^high^ DC after 24 hours (Supplementary Fig. S1a), setting the stage for a kinetics analysis. In the bone marrow, we observed a rapid early drop in NK cell number after 2 days of DC depletion, further decreasing until 6 days (Fig. 1a). In the spleen, DC depletion led to a more gradual reduction of NK cell numbers, reaching approximately 50% of normal levels at day 10 compared with non-depleted mice (Fig. 1a). The fractions of CD11c+ NK cells were similar in CD11c.DOG and littermate controls after DT administration (Supplementary Fig. S1b), suggesting that the overall drop in NK cell numbers reflected an indirect effect of DC depletion and not a direct cytotoxic targeting of CD11c^+^ NK cells by the toxin^10^.

To evaluate if the drop in NK cell number was associated with a change in survival or proliferation of mature splenic NK cells, we investigated markers for apoptosis (Annexin V) and proliferation (Ki67) on the splenic NK cell population during the 10 days of DC depletion. Already 2 days after DT administration, the fraction of Annexin V^+^ NK cells increased approximately 2.5 fold in the NK cell pool (Fig. 1b). It showed a further increase at 4 days after administration and reached a maximum at 8 days. The fraction of Ki67^+^ NK cells increased significantly after the induction of apoptosis 4 days of DC depletion. The peak of proliferation was reached at day 6 (up to 4-fold increase), and proliferation went back to baseline at day 10 (Fig. 1b).

Murine NK cells can be divided into four developmental stages by the markers CD27 and CD11b^18^. The most immature NK cell subset (CD27^low^CD11b^low^) increased after DC depletion, with a peak at 8 days of DC depletion (Fig. 1c,d). In parallel, we noted an abrupt drop in the fraction of CD27^high^CD11b^high^ NK cell subset after 4 days of DC depletion, which remained low until day 10 (Fig. 1c,d). Among the more mature NK cells, there was no change in the frequency of CD27^high^CD11b^low^ NK cell subset during the 10-day period, while the most mature CD27^low^CD11b^high^ NK cell subset tended to increase after DC depletion. We asked if the accumulation of CD27^low^CD11b^low^ NK cell subset resulted from an increased proliferation of these cells in DC-depleted compared to non-depleted mice. We indeed found that this subset made up a larger fraction of Ki67^+^ NK cells in DC-depleted *versus* non-depleted mice, compared to the other subsets (Fig. 1e).

We conclude that the presence of DC was required at steady-state to maintain the balance between survival and proliferation of splenic NK cells, and that perturbing this balance reduced the NK cell numbers and lead to an overall more immature NK cell pool.

### Alteration of NK cell activating and inhibitory receptors in the absence of dendritic cells

**Figure 2:**
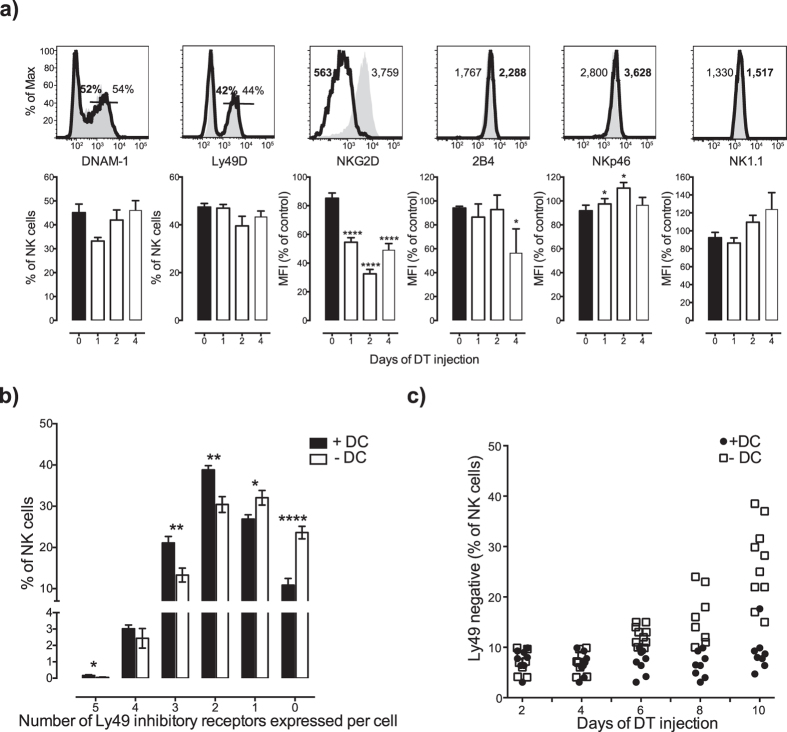
Altered receptor expression on NK cells upon DC depletion. (**a**) Expression of activating receptors on NK cells from the spleen. CD11c.DOG mice (white bars) were injected with DT daily for 1, 2, and 4 days and compared to data from littermate controls (black bar) at day 0. (**b**) Inhibitory receptor repertoire on NK cells upon 10 days of DT injection. Five inhibitory receptors, including Ly49C, Ly49I, Ly49A, Ly49G2, and NKG2A were analyzed. (**c**) Percentages of Ly49-negative subsets (negative for 5 inhibitory receptors tested) upon daily DT injection for 2, 4, 6, 8, or 10 days. *p < 0.05, **p < 0.01, ***p < 0.001, and ****p < 0.0001 (unpaired two-tailed Student’s t-test). Data shown are combined from 3 independent experiments (**a,b,c**). Error bars, SEM (n = 4 to 10 mice per group [A], n = 8 to 10 mice per group [**b**,**c**]).

In the spleen, the activating receptors DNAM-1, Ly49D, 2B4, NKp46 and NK1.1 were largely unaffected by DC depletion (Fig. 2a). In contrast, the NKG2D receptor was strongly downregulated already one day after the onset of DT administration and remained low after 4 days of DC depletion in both spleen (Fig. 2a) and BM (data not shown). We then examined the frequency of NK cells expressing the most commonly expressed inhibitory receptors in B6 mice, namely Ly49C, Ly49I, Ly49A, Ly49G2, and NKG2A. These receptors divide the NK cell population into 32 subsets^19^,^20^. When the frequencies of these subsets were compared between wild type and DC-depleted mice, the most striking alteration was an increase in the frequency of NK cells lacking all five receptors (Fig. 2b), but we also found a small increase in NK cells expressing one receptor and a significant decrease in NK cell subsets expression 2, 3, 4 or 5 receptors (Fig. 2b). A kinetic analysis revealed that the accumulation of Ly49 receptor-negative NK cells started at 6 days after DT administration, and increased with time (Fig. 2c).

### DC depletion results in reduced NK cell function independent from the effects on NK cell maturation

**Figure 3:**
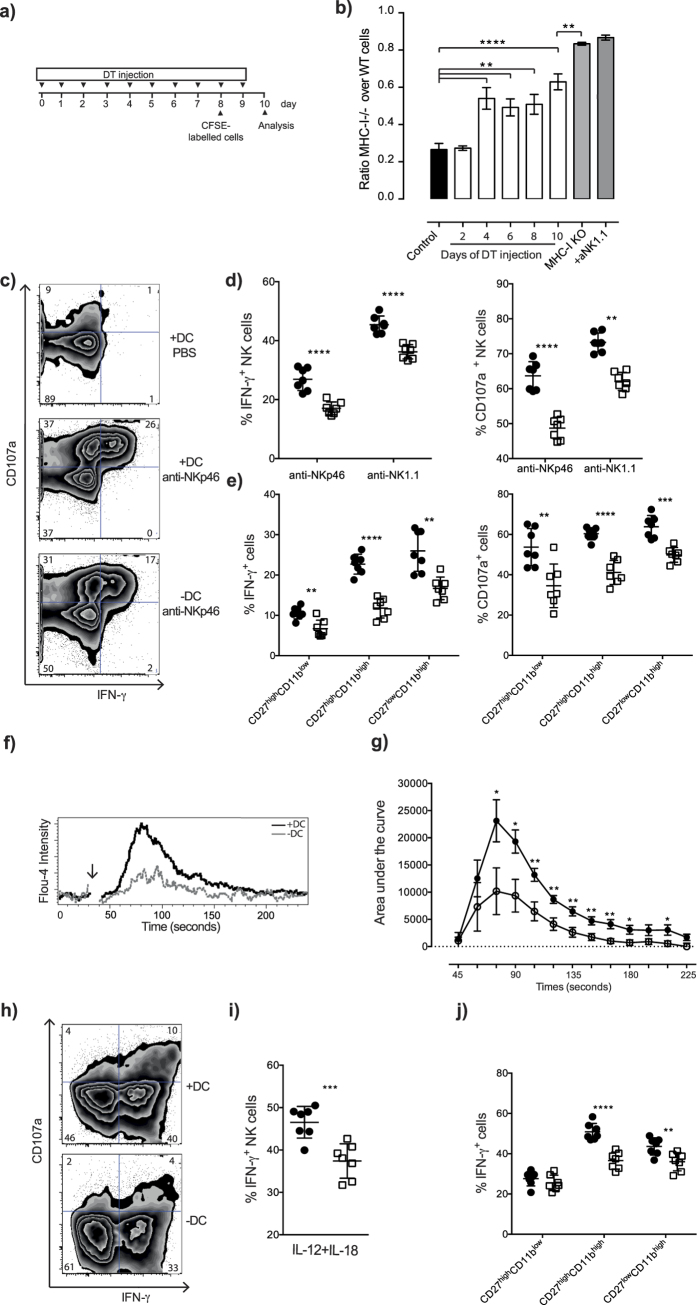
Dendritic cells are required for NK-cell effector functions *in vivo* and *in vitro.* (**a**) Experimental scheme for CFSE-based *in vivo* rejection assay. (**b**) Kinetic of *in-vivo* survival of MHC-class I-deficient target cells. (**c–e**) *In vitro* activating receptor stimulation of NK cells from mice after 4 days of DT injection. (Filled circles) littermate controls, (Empty squares) CD11c.DOG. (**c**) Representative plots of IFN-γ and CD107a expression after anti-NKp46 stimulation. (**d**) Summary of IFN-γ and CD107a expression on NK cells after anti-NKp46 and anti-NK1.1 stimulation. (**e**) Summary of IFN-γ and CD107a expression on NK cells of different maturation stages upon anti-NKp46 stimulation. (**f,g**) Ca^2+^ mobilization upon activating receptor stimulation after 4 days of DT injection. (**f**) Representative plots showing the calcium flux response of NK cells after stimulation of the NK1.1 receptor using antibodies and crosslinker. The arrow indicates the time when the crosslinker was added. (**g**) Summary plots of 3 experiments where the area under the curve was determined using values before addition of the crosslinker as a baseline. (**h–j**) Purified NK cells at 4 days after the start of DT injection were stimulated with IL-12 (1 ng/ml) and IL-18 (1 ng/ml). (**h**) Representative plots of IFN-γ and CD107a expression on NK cells upon IL-12 plus IL-18 stimulation. (**i,j**) Summary of IFN-γ expression on NK cells upon IL-12 plus IL-18 stimulation. *p < 0.05, **p < 0.01, ***p < 0.001, and ****p < 0.0001 (unpaired Student’s t test). Data shown are representative of 2 independent experiments (**b**), combined from 2 independent experiments (**d,e,i,j**), combined of 3 independent experiments (**g**). Error bars, SEM (n = 3–4 mice per group [**b**], n = 6–7 mice per group [**g**], n = 7 mice per group [**d,e,i,j**]).

To test if DC depletion affected NK cell function, we first tested the *in vivo* capacity of NK cells from DC-depleted mice to mediate “missing self” rejection using a CFSE-based adoptive transfer setting (Fig. 3a). This assay measures the relative rejection of MHC class I-deficient spleen cells over control cells in the same animal^21^. At two days after DC depletion, “missing self” reactivity was indistinguishable between DC-deficient and DC-sufficient mice, suggesting that NK cell activity was preserved despite DC depletion (Fig. 3b). In contrast, 4 days after DC depletion, “missing self” rejection capacity was reduced by approximately 50% compared to WT mice; a functional defect that persisted up to 10 days of depletion (Fig. 3b). DC-depleted mice were not complete anergic, since they still rejected MHC class I-deficient spleen cells to some extent (Fig. 3b).

The number of NK cells in the spleen was still close to normal at 4 days after DC depletion (Fig. 1a), making it unlikely that lower NK cell numbers could explain the reduced *in vivo* rejection capacity at this time point. Instead, we considered the possibility that individual NK cells had become less functional as a result of DC removal. To test this directly, we stimulated purified NK cells *in vitro* with plate-bound anti-NK1.1 or anti-NKp46 antibodies and analyzed the capacity of NK cells to degranulate and produce IFN-γ. This experiment revealed a lower frequency of NK cells that produced IFN-γ and CD107a in DC-depleted mice compared to non-depleted mice (Fig. 3c,d).

An important question was if the reduced NK cell function was a reflection of a larger proportion of immature NK cells in the spleen (Fig. 1d), or if the effects of DC depletion on NK cell function was independent from NK cell maturation. To differentiate between these possibilities, we included antibodies against maturation markers in our analysis panel. This analysis clearly showed that NK cells from DC-depleted mice were hyporesponsive across maturation stages (Fig. 3e), implying that DC depletion affected NK cell responsiveness in a process independent from the effect on homeostasis.

Intracellular Ca^2+^ mobilization is an early step in NK cell receptor activation (Supplementary Fig. S2a) that lead to NK cell cytotoxicity and cytokine production^22^. Before analyzing the calcium signaling, we confirmed that there was an equal loading with the calcium dye in NK cells from control or DC compromised mice (Supplementary Fig. S2b). Interestingly, NK cells from DC-depleted mice showed an impairment in Ca^2+^ flux when compared to control mice after NK1.1 cross-linking (Fig. 3f,g; Supplementary Fig. S2B), suggesting an early signaling defect in NK cells from DC-depleted mice. We also found that the capacity of NK cells to respond to stimulation with the cytokines IL-12 + IL-18 was compromised in the absence of DC (Fig. 3h,i). Similar to ITAM-mediated signaling, cytokine production in response to cytokine stimulation was also compromised across maturation subsets (Fig. 3j). Overall, our experiments suggest that DC dynamically control NK cell responsiveness in a broad sense, and in a process that is independent from homeostatic effects mediated by DC.

### NK cell control by DC at steady-state is IL-15 dependent

**Figure 4:**
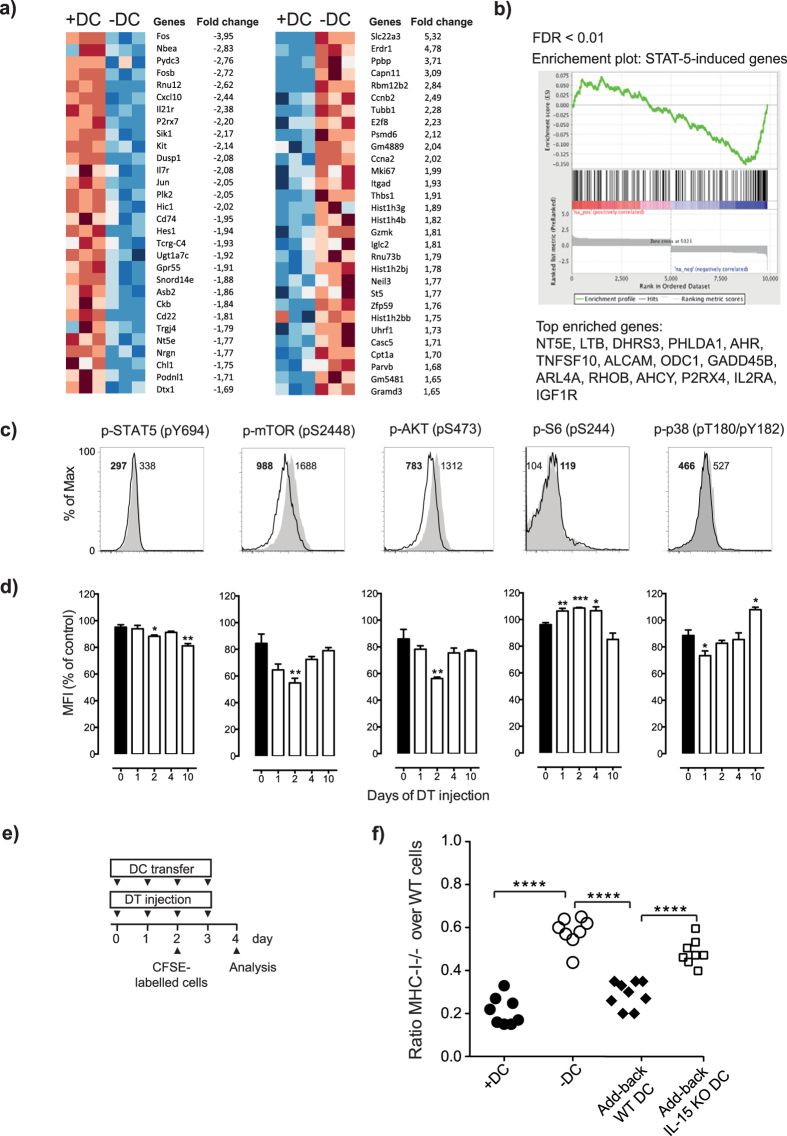
NK cell priming by DC is IL-15-dependent. (**a,b**) Microarray analysis of NK cells after 4 days of DT injection. (**a**) Top 30 differentially expressed genes. Red color shows up-regulated genes and blue color shows down-regulated genes. (**b**) Gene enrichment analysis (GSEA) comparing our ranked genes based on fold changes with the STAT5-induced gene list available at http://software.broadinstitute.org/gsea/, systematic name: M5947. (**c**) Phosphorylation of STAT5, mTOR, AKT, S6, and p38 from freshly-isolated NK cells without any stimulation in the presence or absence of DCs for 2 days. (**d**) Kinetic phosphorylation of IL-15 signaling molecules in NK cells in the presence or absence of DCs for 1, 2, or 4 days. (**e**) Experimental scheme for add-back experiment. (**f**) Summary for the add-back experiment. *p < 0.05, **p < 0.01, ***p < 0.001, and ****p < 0.0001 (unpaired Student’s t test). Data shown are representative from 2 independent experiments (**c**,**d**) and combined of 3 independent experiments (**e,f**). Error bars, SEM (n = 6 per group [**d**], n = 8–9 mice per group [**f**].

To gain understanding of the molecular changes taking place in NK cells upon DC depletion, we performed a microarray gene expression analysis, focusing our analysis on sorted splenic NK cells from mice that had been DC-depleted for 4 days. Setting a threshold of a 1.3-fold change, we found 149 significantly downregulated and 143 upregulated genes in NK cells from DC-depleted *versus* non-depleted mice. Using the DAVID functional annotation tool to cluster genes based on gene ontology (GO) annotations for biological processes, we identified six statistically significant GO terms associated with the immune system that were enriched among the down-regulated genes (Table 1). Altogether, 36 genes contributed to these clusters (Supplementary Table S1). Of those, many have been associated with NK cell function, such as lymphotoxin B (*Ltb*), cytotoxic and regulatory T cell molecule (*Crtam*), CD38 antigen (*Cd38*) and lymphocyte-activation gene 3 (*Lag3*). Interestingly, in the GO term “positive regulation of metabolic process”, we found several genes encoding for transcription factors that are also essential for NK-cell effector functions, including FBJ osteosarcoma oncogene (*Fos*), jun proto-oncogene (*Jun*), proto-oncogene c-kit (*Kit*) and aryl-hydrocarbon receptor (*Ahr*). Many of the genes that contributed to these clusters belonged to the 30 most down-regulated genes (Fig. 4a). Thus, DC depletion had a marked impact at the transcriptional level of genes related to NK cell effector functions, supporting the reduction in function. Regarding upregulated genes, the processes we identified were mostly associated with cell division (Table 1 and Supplementary Table S2), and within the 30 most up-regulated genes, 9 were part of the cluster analysis (Fig. 4a). These data corroborate the start of NK cell proliferation that was evident already at 4 days after DC depletion (Fig. 1b).

One key observation from the gene array analysis was that many of the down-regulated genes are known to be regulated by IL-15, such as *Fos*, *Jun*, *Kit*, CXCL10 and IL-21 receptor (*Il21r*) (Fig. 4a). Interestingly, a gene set enrichment analysis (GSEA) of our gene array data with a previously published signal transduction and activation of transcription (STAT)-5-induced reference gene set, known to be controlled by IL-15, showed a significant enrichment with down-regulated genes in NK cells isolated from DC-depleted mice (Fig. 4b).

To further follow this lead, we investigated intracellular pathways of IL-15R signaling in NK cells from DC-depleted mice. IL-15R signaling involves three different pathways, including, mitogen activated protein kinase, and PI3K-AKT-mTOR^6^. Addition of IL-15 to highly purified NK cells induced an increase in the phosphorylation of STAT-5, mTOR, Akt, p38 and S6 molecules confirming previously published data (Supplementary Fig. S3). In line with this pathway of control, with the exception of S6, we found reduced phosphorylation of these proteins in NK cells from DC-depleted mice compared to control mice (Fig. 4c,d). For STAT5, Akt and mTOR, the strongest reduction was observed at day 2 after the onset of DC depletion. In contrast, the early significant reduction of phospho-p38 was observed at day 1 after DC depletion. Together, this data confirm our conclusion from the gene array data and indicate that DC depletion leads to a rapid down-regulation of IL-15R signaling pathways in NK cells.

Because we observed a faster down-regulation of NKG2D in the surface of NK cells after DC depletion, we decided to test directly the link between NKG2D expression and IL-15 stimulation. We thus cultured NK cells in the presence or absence of IL-15 and the cell surface marker NKG2D was analyzed 8 hours later (Supplementart Fig. S4). An increase in the expression of NKG2D at the surface of mouse NK cells was seen after addition of IL-15 (Supplementary Fig. S4a). Similar findings were also seen on human NK cells (Supplementary Fig. S4b). These experiments also revealed that IL-15 starvation resulted in a down-regulation of the NKG2D expression on mouse and human NK cells in the absence of IL-15 (Supplementary Fig. S4a,b). On the other hand, DNAM-1 and NKp44 expression did not change following 36-hour culture (data not shown). To evaluate the contribution of DC-mediated IL-15 presentation in the maintenance of the NKG2D expression, human NK cells were cultured alone, treated with IL-15, together with DCs, or with DCs and IL-15 for 36 hours. Upon co-culture with DC, human NK cells maintained NKG2D expression compared to newly isolated NK cells (Supplementary Fig. S4c, compare group “NK alone” and “NK-DC”). In addition, NK cells cultured with IL-15 showed an increase in the expression compared to controls and further culture with DC and IL-15 revealed a synergistic effect on NKG2D expression on NK cells (Supplementary Fig. S4c). Taken together, these findings support the notion that IL-15 facilitates NKG2D expression, in particular in the presence of DCs.

Finally, to test if IL-15 signaling by DCs was important to maintain NK cell “missing self” function *in vivo*, we performed experiments in which IL-15-sufficient or IL-15-deficient DC (from mice lacking the DTR transgene) were co-administered with DT for 4 days to CD11c.DOG mice (Fig. 4e). Recipient mice would thus be depleted from endogenous DCs (DTR+) but not from the added DCs (DTR−). As a source of DC for these adoptive transfer experiments, we used spleen cells from B6 mice injected with Flt3L 10 days before, to expand DC precursors and mature DC in the spleen^23^. Cells from the spleens of Flt3L-treated mice were further enriched using CD11c beads. The cells that were added back were all CD11c^high^ and MHC class II+. Sixty percent also expressed CD8 (data not shown), confirming a high proportion of DC capable of trans-presenting IL-15^24^,^25^.

These experiments conclusively showed that “missing self” function could be restored in the add-back group with IL-15-sufficient DC with a killing response almost similar to WT mice (Fig. 4f). In contrast, neither add-back of similarly processed IL-15-deficient DC, nor of IL-15-sufficient B cells (data not shown), restored NK cell function (Fig. 4f). Altogether, these data suggest that IL-15 is a key player in DC-mediated control of basal NK cell responsiveness, in the absence of inflammation.

## Discussion

Previous investigations have shown that human and murine NK cell exhibit contacts with DC under steady state conditions and in various tissues^12^,^13^,^26^. It has been proposed that one possible reason for NK cells to contact DC *in vivo* is to interact with *trans*-presented IL-15, which is required for normal NK cell homeostasis^10^. This happens also during inflammatory conditions and infections, when IL-15 from DC is required for NK cell priming in lymph nodes^5^. Studies of NK cell interactions with DC *in vivo* have revealed that contacts between these cells are both multiple and transient^13^. It has been estimated that NK cells spend on average 40–60% of their time in contact with DC in lymph nodes, both during activation and at steady state^13^. The significance of this interesting finding has not been clarified.

Our finding that several IL-15-controlled events, such as NKG2D cell surface expression and phosphorylation of mTOR and AKT, were perturbed in NK cells already at one day after the onset of DC depletion, suggest that IL-15 *trans*-presentation induce only transient responses in naïve NK cells. The many short and frequent interactions that are seen between NK cells and DC *in vivo*^13^ may represent an adaptation to this property of IL-15 control, securing continuous interactions with DC to maintain a functional NK cell pool. Why such a mechanisms would be required to maintain NK cell functionality is an interesting question. One possibility is that it would reflect a need for NK cells to constantly re-evaluate their environment for changes in self markers, to adjust their responsiveness. We have proposed a “rheostat” model for such a regulation of NK cell responses, which is sensitive to changes in the MHC class I setup of surrounding cells^19^,^20^,^27^. We have suggested a role for IL-15 as a regulator of this process^19^, but more work is needed to investigate the possible link between NK cell education and the regulation of NK cell homeostasis we observed in this study.

The kinetics of the effects on the NK cell pool is of interest. Taking into account the quite long time it takes before most DC have been depleted after DT administration (at least 12 hours), we conclude that that no more than 10–12 hours need to pass before the initial effects of continuous IL-15 triggering ceases. It is likely that this time is much shorter, at least for the mTOR and AKT phosphorylation^28^, but more kinetic studies are required. With regard to these early molecular events, we noted that the steady-state phosphorylation of a third major signaling protein, S6, was not altered upon DC deletion. This finding might be related to recently identified IL-15-independent pathway for S6 phosporylation^29^. Our *in vitro* findings of a link between NKG2D expression and IL-15 treatment, coupled to previously published data in the same direction by others^26^,^30^,^31^,^32^, lead us to favour a early IL-15 withdrawal as an explanation for reduced NKG2D expression. Increased levels of soluble NKG2D ligands as a consequence of DC-depletion might be one contributing reason^33^. Additional work might shed light on this possibility

In parallel to the early molecular events, we noted functional and phenotypic changes in the NK cell pool following DT administration. We did not analyse the mice for those properties earlier than 2 days after the onset of DT administration, which precludes determination of how these homeostatic effects are related to the early molecular findings. Nevertheless, the first indication of a homeostatic change was the apperance of a fraction of apoptotic NK cells in the spleen at 2 days after the onset of DT administration. In parallel, we noted a reduction in NK cell numbers, corroborating previous studies showing loss of mature NK cells after the ablation of CD11c^high^ cells in chimeric mice^34^, and failed survival of adoptively transferred NK cells in DC-depleted lymphopenic mice^10^. Interestingly, loss of NK cells was more pronounced in the bone marrow, suggesting that NK cells at this site may be particularly dependent on DC-derived IL-15. The link between the onset of apoptosis and the loss of NK cells in our data is consistent with a role for IL-15 as a survival factor^35^.

It was more suprising to note an increase in the proliferation of NK cells starting at 4 days after DT administration. Proliferation was mainly seen in immature NK cells (CD27^low^CD11b^low^), but also more mature NK cells (CD27^high^CD11b^high^) proliferated to some extent^36^. A dramatic change was the accumulation of Ly49 receptor negative NK cells, which is also a phenotype of immature NK cells. DC were previously shown to affect the differentiation of sorted Ly49-receptor-null NK cells to Ly49-positive NK cells^37^, suggesting that DC may promote Ly49 receptor expression as such. It is unclear if the DC effect we observe *in vivo* is related to such a mechanism, or whether it is mediated primarily via homeostatic mechanisms. The latter is perhaps more likely, as the change in composition of the Ly49 receptor repertoire closely followed the onset of proliferation of immature NK cells.

Because DC are continuously depleted for 10 days in our model, factors driving this proliferative burst must come from other cells. Neutrophils represent one interesting candidate. These cells accumulate in the spleen of DC-depleted mice and could provide a new source of IL-15 to NK cells in DC-depleted mice. In fact, when neutrohils were depleted from DC-depleted mice, we observed a synergistic and accelerated loss of NK cells (data not shown), suggesting that neutrophils indeed might compensate for lack of IL-15 from DC^38^. Another alternative might be stromal cells, which provide a major source of IL-15 *in vivo*^39^. However, we do not know if IL-15 might still be the primary cytokine driving NK cell proliferation, and it is possible that other factors could substitute, such as IL-12 or IL-2 coming from other sources^40^. We can at least conclude that whathever factors that drive proliferation, it acts primarly on immature NK cells and can not restore function that has been lost by DC depletion. Further studies could address if some subsets of NK cells are particularly dependent on the DC crosstalk, which might lead to new ways by which the size of certain subsets can be influenced clinically.

Our DC add-back experiment suggested that the IL-15 trans-presenting cells that provide signals to NK cells *in vivo* are classical IL-15-expressing CD11c + MHC class II + DC (likely also CD8+), but further than that, our study does not address the phenotypes of the DC subsets involved. This important question could potentially be addressed using new genetic models, allowing conditional depletions and adoptive transfer of subsets of DC^41^.

It was of particular interest in our study that the decrease of NK cell responsiveness following DC disapperance was seen across stimulatory pathways, from “missing self” reponses *in vivo* to cytokine production and degranulation *in vitro* in response to triggering of specific ITAM-controlled activating receptors and cytokine receptors. Importantly, this loss was not associated with the alteration of NK cell maturation, as functional deficiency occurred in all maturation stages. The simplest explanation is that early IL-15 signaling controls key and general components required to alert the NK cell to respond. Our data that early calcium flux after triggering of the ITAM-coupled receptor NK1.1 was compromized suggest that such control is exerted at the level of early signaling, but more work is required to clarify this interplay.

Our study significantly expand our understanding of an important continuous delivery of IL-15 signal by DC to control NK cell responsiveness, not only during inflammation^5^ but also at steady-state. It might be interesting to consider these findings in the context of previously published data on T cells after DC depletion. Indeed, our data are reminiscent of the loss of antigen sensitivity of naïve T cells to foreign antigens that has been observed after DC depletion, which may suggest similarities in how these cell types are regulated by external signals^3^,^4^. Identifying additional player involved in the control of NK cells by DC is an important next step. Here, comparisons of the non-responsiveness observed in NK cells early after DC depletion to NK cells in MHC-I-deficient animals/individuals may be informative. For example, whether or not interactions with MHC class I is involved in NK cell intereactions with DC warrants further studies, Finally, while we favour a direct role of the DC/NK cell crosstalk in control of NK cell homeostasis and function, we can not at this stage exclude the possibility that DC exert their NK cell control via additional cell types that may be affected by DC depletion such as neutrophils, which increase in numbers following DC depletion. Further work will have to be designed to specifically test this possibility.

## Methods

### Regulations

All experimental procedures in this study were performed in accordance with guidelines and regulations at Karolinska Institutet and the Karolinska University Hospital. All animal experiments were approved by the Stockholm branch of the national ethics committee in Sweden (Stockholms Södra Djurförsöksetiska Nämnd).

### Mice and cell lines

Mice were bred and maintained in the animal facility at Karolinska Institute, Huddinge, Sweden. Experimental mice were at the age of 8–12 wks. C57BL/6 J mice (B6, CD45.1^+^) were purchased from Jackson Laboratory. CD45.2^+^ B6 mice were purchased from Charles River. IL-15 deficient mice^42^ were purchased from Taconic laboratory. B6. CD11c.DOG mice were obtained from Heidelberg, Germany^10^.

### Antibodies and flow cytometery analysis

Splenocytes in a single-cell suspension were incubated with anti-FcγRIII (2.4G2) before surface marker staining. All surface staining was done at 4 °C in PBS supplemented with 2% FBS for 20 minutes. Antibodies that were used in this study are CD3 (145-2C11), KLRG1 (2F1) from eBiosciences, NK1.1 (PK136), DX5 (DX5), CD27 (LG.3A10), Ly49A (YE1/48.10.6), NKG2A (16I11), Gr-1 (RB6-8C5), CD4 (GK1.5), CD8α (53–6.7), CD45.1 (A20), CD45.2 (104), DNAM-1 (480.1), Ly6G (A18), Ly6C (HK1.4), CD11c (N418), CD122 (5H4), CD117 (2B8), and CD19 (6D5) from BioLegend, NKp46 (29A1.4), CD11b (M1/70), Ly49I (YLI-90), Ly49G2 (4D11), Ki67 (B56), IFN-γ (XMG 1.2), I-A[b] (AF6-120.1), CD127 (A7R34), and Flt3L (A2F10.1) from BD Pharmingen (Stockholm, Sweden). Anti-Ly49C (4LO3311) hybridoma was a kind gift from Suzanne Lemieux (INRS–Institut Armand-Frappier, Laval, Quebec). Anti-Ly49C antibodies were biotinylated following DSB-X™ Biotin Protein Labeling Kit (Life Technologies). Dead cells were excluded using LIVE/DEAD^®^ Fixable Aqua Dead Cell Stain Kit (Invitrogen). Ki67 staining was done using Foxp3/Transcription Factor Staining Buffer Set (eBiosciences). Annexin V staining was performed in Annexin V binding buffer (BD Pharmingen). Following isolation and co-cultures, human NK cells were stained with fluorochrome-conjugated antibodies against CD56, CD3, NKG2D, DNAM-1, NKp44, and live/dead cell dye (Biolegend, San Diego, USA and Invitrogen, Oregon, USA) and DCs (data not shown) for CD14, CD11C, HLA-DR, CD83, and live/dead cell dye, according to the manufacturing.

### *In vitro* stimulation assay

24-well plates were coated overnight at 4 °C with 20 μg/ml anti-NK1.1 (PK136, eBiosciences), anti-Ly49D (4E5, BD Pharmingen), anti-NKp46 (polyclonal, R&D systems). The day after, splenocytes were prepared and NK cells were enriched using negative selection kit (Invitrogen). NK cells (4 × 10^5^ to 5 × 10^5^ cells) were stimulated for 6 hours at 37 °C. CD107a (BD Pharmingen) was added (1:300 dilution) to the culture during the stimulation. After the first hour, GolgiPlug™ Protein Transport Inhibitor (BD Pharmigen) was dispensed into the culture. 1 μg/ml of phorbol-12-myristate-13-acetate (PMA) plus 0.25 μg/ml of ionomycin was used for positive control and PBS alone for negative control. For cytokine stimulation, IL-18 (1 ng/ml) plus IL-12 (1 ng/ml,) was used. After 6 hours, cells were stained with Fc block and surface antibodies as described above. For the intracellular staining, cells were fixed and then permeabilized using Cytofix/Cytoperm kit (BD Pharmingen), followed by staining with anti-IFN-γ (XMG1.2).

### Enrichment of DCs and NK cells from peripheral blood

DCs were isolated from healthy donors as previously described^43^. Briefly, peripheral blood mononuclear cells (PBMC) were washed and allowed to adhere into plastic culture 10-cm Petri dishes at a concentration of 2–5 × 10^6^/ml in serum-free Dulbecco’s modified Eagle’s minimal essential medium for 2 hours at 37 °C. Non-adherent cells were washed away and adherent cells were further cultured for 18 hours in DMEM medium supplemented with 2 mM L-glutamine, 1% non-essential amino acids and 5% FCS. Following overnight (18-h), dishes were shaken for 20 min by an orbital shaker, and refloating cells (DC-enriched fraction) were collected and counted. Autologous NK cells were isolated from PBMC by immunomagnetic depletion (Miltenyi Biotech) and plated in 24-well (0.25–0.5 × 10^6^ per well) in DMEM media supplemented with 5% FCS. DCs were added to NK cells at 1:1 ratio in presence or absence of recombinant human IL-15 (10–100 ng/ml, PeproTech, London, UK) for 4–36 hours.

### Phosphoflow experiments

Murine splenic NK cells were sorted using BD FACSAria^TM^III (more than 95% purity). Sorted cells were stimulated with IL-15 (100 ng/ml) for 20 min at 37 °C. The stimulation was attenuated by fixation with BD Cytofix™ - Fixation Buffer (BD Pharmingen) for 10 min at 37 °C. Cells were then permeabilized by dropwise adding Perm Buffer III (BD Pharmingen) and incubated for 30 min on ice. The staining with phosphoflow antibodies was done at room temperature for up to one hour. Antibodies (BD Pharmingen) used to detect phosphorylated signaling molecules are anti-p-STAT4 (pY693), anti-p-STAT5 (pY694), anti-p-p38 (pT180/pY182), anti-p-ERK (pT202/pY204), anti-p-AKT (pT308), anti-p-AKT (pS473), anti-p-mTOR (pS2448), anti-4-EBP1 FITC (pT69), and anti-pS6 PE (pS244).

### Calcium flux experiment

Freshly purified NK cells from DT-injected CD11c.DOG (day 4) and littermate controls were incubated *in vitro* with purified mAb against NK1.1. Antibody-coated NK cells were stained with calcium dye Fluo-4 (Life Technologies) and purified Goat-Anti-Mouse IgG (H + L) Polyclonal Antibody (Jackson Immunoresearch) was added as indicated in the figure for measurement of real-time iCa^2+^ mobilization by flow cytometry. Receptor induced iCa^2+^ mobilization was measured by determining the intensity of iCa^2+^ peak and the integration of iCa^2+^ over time.

### *In vivo* rejection and transfer assay

To obtain DCs for add-back experiments, 10 μg of human Flt3L (BioXCell) was injected subcutaneously daily for 7–9 days to wild type or IL-15-deficient mice. CD11c.DOG mice and their littermate controls were injected with DT (8 ng/g body weight) (Sigma Aldrich) intraperitoneally daily for 4 days. DC were isolated from Flt3L-injected mice using CD11c microbeads (Miltenyi Biotec). Ten million IL-15 deficient or sufficient DCs (CD11c^high^ MHC class II+) were transferred daily during 4 days. At day 2, splenocytes from wildtype and MHC^−/−^ mice were labeled with 5(6)-carboxyfluorescein diacetate N-succinimidyl ester (CFSE; Invitrogen) at 1 μM (CFSE^low^) and 10 μM (CFSE^high^) and mixed at a ratio of 1:1. Subsequently, 10 × 10^6^ cells from the mixture were injected intravenously into CD11c.DOG mice and littermates. In this assay, the relative rejection of MHC class I-deficient cells can be determined by comparing the ratio of CFSE^low^ and CFSE^high^ cells in the spleens of the responder mice. A group of WT mice was injected with anti-NK1.1-depleting antibody 2 days before injection of CFSE-labeled cells. Two days later, flow cytometry was performed to analyze the survival of target cells. NK cell phenotype characterization and proliferation analysis were carried out in some experiments.

### Microarray analysis

Splenic NK cells were sorted using BD AriaIII (BD Biosciences). Total RNA was extracted from cell lysate and loaded onto The Affymetrix^®^ Mouse Gene 1.1 ST Array (operated by BEA core facility, Dep. of Biosciences and Nutrition, Karolinska Institutet, Huddinge). Data were normalized and analyzed using R programming. Gene set enrichment analysis were done using DAVID online software (https://david.ncifcrf.gov) and GSEA software.

## Additional Information

**How to cite this article**: Luu, T. T. *et al*. Independent control of natural killer cell responsiveness and homeostasis at steady-state by CD11c+ dendritic cells. *Sci. Rep.* **6**, 37996; doi: 10.1038/srep37996 (2016).

**Publisher's note:** Springer Nature remains neutral with regard to jurisdictional claims in published maps and institutional affiliations.

## Supplementary Material

Supplemental Material

## Figures and Tables

**Table 1:** Functional biological process-enrichment analysis of differentially expressed genes (1.3 folds).

Rank	Biological process^*a*^	Enrichment^*b*^ (Fold)	*P* value^*c*^	*Genes present*
***Processes enriched for down-regulated genes***(***149 genes***)				
1	Immune response	3.8	1.4E-4	Cd74, Ang4, Ccl4, Cxcl10, Crtam, Lilrb4, Gp49a, H2-Ab1, Il4ra, Il7r, Ltb, Ncf1, Ccl27a, Tnfsf10
2	Regulation of cell killing	18	1.3E-3	Crtam, Il7r, Lag3, P2rx7
3	Positive regulation of immune system process	4.7	3.7E-3	Cd38, Cd74, Il4ra, Crtam, Il7r, Lag3, P2rx7
5	Lipid transport	5.8	1.1E-2	Atp8a2, Cpt1b, Pltp, Kcnn4, P2rx7
6	Programmed cell death	2.6	2.1E-2	Ahr, Kit, Ltb, Ncf1, Pmaip1, Phlda1, Mrpl41, Ppp1r15a, Tnfsf10
7	Positive regulation of macromolecule metabolic process	2.2	3.9E-2	Bcl11b, Cited4, Fos, Jun, Ahr, Hes1, Kit, Ltb, Nr4a2, P2rx7
***Processes enriched for up-regulated genes***(***143 genes***)				
1	DNA packaging	18	8.6E-13	Asf1b, H2afz, Hells, Hist1h1a, Hist1h1b, Hist1h2ab, Hist1h2bb, Hist1h2bc, Hist1h2bh, Hist1h3g, Hist1h4h, Hist1h4b, Hist1h4c, Hist2h4, Hist1h4f, Hist4h4, Hist2h2ab, Nusap1, Hist1h2bj
2	Cell division	8.6	5.2E-12	Anln, Aspm, Aurkb, Bub1, Bub1b, Cdca7, Cdca8, Cenpe, Ccna2, Ccnb2, Ccne2, Hells, Kif11, Lig1, Nusap1, Plk1, Cks1b, Sgol2, Ube2c
3	Microtubule-based movement	10	1.4E-5	Cenpe, Kif11, Kif15, Kif18b, Kif20a, Kif23, Kif4, Tubb1
4	DNA metabolic process	3.6	4.4E-4	Prim1, Rad51, Clspn, Ccne2, Exo1, Hells, Lig1, Mcm7, Neil3, Pole, Rrm2, Uhrf1
5	Cell proliferation	4.1	3.2E-3	E2f8, Mki67, Aspm, Hells, Itgad, Mcm7, Vpreb1, Uhrf1

^a^Gene ontology for biological processes containing down-regulated genes and up-regulated genes in NK cells from DC-depleted mice for four days enriched relative to presentation that would occur by chance.

^b,c^Enrichment and *P* values (from a modified Fisher’s exact test) were calculated with David online software.
