# Supplementary material for: Independent control of natural killer cell responsiveness and homeostasis at steady-state by CD11c+ dendritic cells
Source: Sci Rep. 2016 Dec 1;6:37996. doi: 10.1038/srep37996 (PMC5131354; doi:10.1038/srep37996)
Supplement: Supplemental Material [file srep37996-s1.doc]

*Luu et al., supplementary material (6 pages)*

*Ms tittle: Independent control of natural killer cell responsiveness and homeostasis at steady-state by CD11c+ dendritic cells.*

*Authors: Thuy Thanh Luu, Sridharan Ganesan, Arnika Kathleen Wagner, Dhifaf Sarhan, Stephan Meinke, Natalio Garbi, Günter Hämmerling, Evren Alici, Klas Kärre, Benedict J. Chambers, Petter Höglund^*^, Nadir Kadri^*^*

**­­­­­**

**Supplementary Figure S1:** DC-depletion removes CD11C+MHC class II+ DC but does not reduce the frequency of CD11c+ NK cells in the spleen. (a) Staining for CD11c+MHC class II+ DC in the spleen of CD11c.DOG and littermate control mice (WT) injected with DT 24 hours before. (B) Expression of CD11c on NK cells from WT and CD11c.DOG mice after 4 days of DT treatment.

**Supplementary Figure S2.** a) Calcium flux via the NK1.1 receptor requires both a primary anti-NK1.1 antibody and addition of a secondary rat anti-mouse crosslinking antibody (black line). Primary antibody alone did not trigger calcium flux (grey line). WT B6 mice were analyzed and PBS or crosslinker were added at the arrow. b) Addition of Ionomycin results in equally efficient calcium flux in DC-depleted and non-depleted CD11c.DOG mice.

**
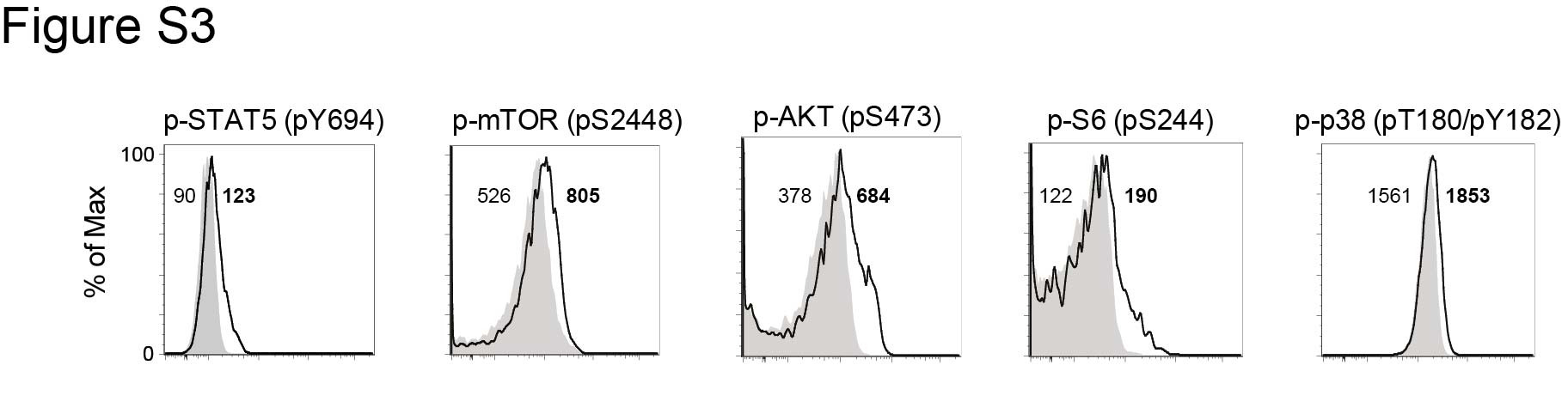
**

**Supplementary Figure S3.** IL-15 stimulation (open histograms) for 20 minutes induced phosphorylation of STAT5, mTOR, AKT, S6 and p38 in NK cells from WT B6 mice. Grey filled histograms represent non-stimulated NK cells.

**Supplementary Figure S4.** Expression of NKG2D on human and mouse NK cells is controlled both by DC and IL-15. (a) NKG2D expression on purified WT B6 NK cells decreased after 8 hours of culture and was induced by IL-15. (b) Similar experiment as in A but with human NK cells. Culture time was 36 hours. (c) The presence of DC prevents loss of NKG2D expression and potentiates the effect of IL-15 on NKG2D induction.

**Supplementary T**able 1: Functional pathway-enrichment analysis of downregulated genes

| Biological process | Genes present |
| --- | --- |
| Immune response | Cd74, Ang4, Ccl4, Cxcl10, Crtam, Lilrb4, Gp49a, H2-Ab1, Il4ra, Il7r, Ltb, Ncf1, Ccl27a, Tnfsf10 |
| Regulation of cell killing | Crtam, Il7r, Lag3, P2rx7 |
| Positive regulation of immune system process | Cd38, Cd74, Il4ra, Crtam, Il7r, Lag3, P2rx7 |
| Programmed cell death | Ahr, Kit, Ltb, Ncf1, Pmaip1, Phlda1, Mrpl41, Ppp1r15a, Tnfsf10 |
| Positive regulation of macromolecule metabolic process | Bcl11b, Cited4, Fos, Jun, Ahr, Hes1, Kit, Ltb, Nr4a2, P2rx7 |
| Lipid transport | Atp8a2, Cpt1b, Pltp, Kcnn4, P2rx7 |

**Supplementary T**able 2: Functional pathway-enrichment analysis of upregulated genes

| Biological process | Genes present |
| --- | --- |
| Cell division | Anln, Aspm, Aurkb, Bub1, Bub1b, Cdca7, Cdca8, Cenpe, Ccna2, Ccnb2, Ccne2, Hells, Kif11, Lig1, Nusap1, Plk1, Cks1b, Sgol2, Ube2c |
| DNA packaging | Asf1b, H2afz, Hells, Hist1h1a, Hist1h1b, Hist1h2ab, Hist1h2bb, Hist1h2bc, Hist1h2bh, Hist1h3g, Hist1h4h, Hist1h4b, Hist1h4c, Hist2h4, Hist1h4f, Hist4h4, Hist2h2ab, Nusap1, Hist1h2bj |
| Microtubule-based movement | Cenpe, Kif11, Kif15, Kif18b, Kif20a, Kif23, Kif4, Tubb1 |
| DNA metabolic process | Prim1, Rad51, Clspn, Ccne2, Exo1, Hells, Lig1, Mcm7, Neil3, Pole, Rrm2, Uhrf1 |
| Cell proliferation | E2f8, Mki67, Aspm, Hells, Itgad, Mcm7, Vpreb1, Uhrf1 |
